# Carotenoids in Cancer Metastasis—Status Quo and Outlook

**DOI:** 10.3390/biom10121653

**Published:** 2020-12-10

**Authors:** Lenka Koklesova, Alena Liskova, Marek Samec, Kevin Zhai, Mariam Abotaleb, Milad Ashrafizadeh, Aranka Brockmueller, Mehdi Shakibaei, Kamil Biringer, Ondrej Bugos, Masoud Najafi, Olga Golubnitschaja, Dietrich Büsselberg, Peter Kubatka

**Affiliations:** 1 Department of Obstetrics and Gynecology, Jessenius Faculty of Medicine, Comenius University in Bratislava, 03601 Martin, Slovakia; koklesova.lenka@gmail.com (L.K.); alenka.liskova@gmail.com (A.L.); marek.samec@gmail.com (M.S.); kamil.biringer@uniba.sk (K.B.); 2 Department of Physiology and Biophysics, Weill Cornell Medicine in Qatar, Education City, Qatar Foundation, 24144 Doha, Qatar; kez4003@qatar-med.cornell.edu (K.Z.); mariam.abotaleb@aucegypt.edu (M.A.); 3 Faculty of Engineering and Natural Sciences, Sabanci University, Orta Mahalle, Üniversite Caddesi No. 27, Orhanlı, Tuzla, 34956 Istanbul, Turkey; dvm.milad1994@gmail.com; 4 Sabanci University Nanotechnology Research and Application Center (SUNUM), Tuzla, 34956 Istanbul, Turkey; 5 Musculoskeletal Research Group and Tumor Biology, Chair of Vegetative Anatomy, Institute of Anatomy, Faculty of Medicine, Ludwig-Maximilian-University Munich, D-80336 Munich, Germany; Aranka.Brockmueller@med.uni-muenchen.de (A.B.); mehdi.shakibaei@med.uni-muenchen.de (M.S.); 6 Lambda Life JSC., 85101 Bratislava, Slovakia; bugos.ondrej@lambda.sk; 7 Medical Technology Research Center, Institute of Health Technology, Kermanshah University of Medical Sciences, Kermanshah 67146, Iran; najafi_ma@yahoo.com; 8 Radiology and Nuclear Medicine Department, School of Paramedical Sciences, Kermanshah University of Medical Sciences, Kermanshah 67146, Iran; 9 Predictive, Preventive, Personalised (3P) Medicine, Department of Radiation Oncology, University Hospital Bonn, Rheinische Friedrich-Wilhelms-Universität Bonn, 53127 Bonn, Germany; Olga.Golubnitschaja@ukbonn.de; 10 Department of Medical Biology, Jessenius Faculty of Medicine, Comenius University in Bratislava, 03601 Martin, Slovakia

**Keywords:** carotenoids, carotenes, xanthophylls, apocarotenoids, metastasis, migration, invasion, epithelial-mesenchymal transition, cancer, patient stratification, individualized patient profiling, liquid biopsy, multi-level diagnostics, artificial intelligence, predictive diagnosis, targeted prevention, personalization of medical services, 3P medicine

## Abstract

Metastasis represents a major obstacle in cancer treatment and the leading cause of cancer-related deaths. Therefore, the identification of compounds targeting the multi-step and complex process of metastasis could improve outcomes in the management of cancer patients. Carotenoids are naturally occurring pigments with a plethora of biological activities. Carotenoids exert a potent anti-cancer capacity in various cancer models in vitro and in vivo, mediated by the modulation of signaling pathways involved in the migration and invasion of cancer cells and metastatic progression, including key regulators of the epithelial–mesenchymal transition and regulatory molecules, such as matrix metalloproteinases (MMPs), tissue inhibitors of metalloproteinases (TIMPs), urokinase plasminogen activator (uPA) and its receptor (uPAR), hypoxia-inducible factor-1α (HIF-1α), and others. Moreover, carotenoids modulate the expression of genes associated with cancer progression and inflammatory processes as key mediators of the complex process involved in metastasis. Nevertheless, due to the predominantly preclinical nature of the known anti-tumor effects of carotenoids, and unclear results from certain carotenoids in specific cancer types and/or specific parts of the population, a precise analysis of the anti-cancer effects of carotenoids is essential. The identification of carotenoids as effective compounds targeting the complex process of cancer progression could improve the outcomes of advanced cancer patients.

## 1. Introduction

Metastasis is a complex process involving the formation of secondary tumors adjacent to or distant from the primary cancer site. The metastatic processes’ mechanisms are complicated and involve multiple signaling molecules and pathways [1]. As one of the hallmarks of cancer [2,3], metastasis is often the cause of treatment failure [1]. In addition, patients receiving chemotherapy often acquire drug resistance, which eventually leads to cancer recurrence and metastasis [4]. Moreover, metastasis constitutes the primary cause of cancer-related death in more than 90% of cancer patients [2]. Although the prevention or early diagnosis of cancer certainly improves clinical outcomes [5], cancer is diagnosed frequently in advanced stages associated with metastasis [6]. Thus, the early detection of metastatic cancer and appropriate therapeutic interventions are key considerations in managing advanced cancer patients [3]. The main obstacle of current treatment modalities is the heterogeneity of metastasis associated with the difference of metastatic tumors from the primary tumor, as well as additional alterations at both the genetic and epigenetic levels. Understanding the precise dynamics of metastatic processes will support the identification of molecular targets potentially applicable in advanced stage cancer patients [2].

Phytochemicals are a class of biologically active compounds with anti-cancer efficacy [4]. The anti-cancer effects of naturally occurring phytochemicals are currently widely discussed [4,7,8,9,10,11,12,13,14]. Importantly, phytochemicals exert a great capacity to reverse or retard metastatic processes and prevent tissue invasion and metastasis [4,15]. Carotenoids are liposoluble pigments often present in orange, yellow, or red vegetables and fruit, as well as fungi, bacteria, and algae [16,17]. Carotenoids have various biological, especially oncologic, effects [18,19,20]. Thus, in this review, we discuss the potentially beneficial effects of carotenoids through the modulation of multistep metastatic processes and the regulation of multiple signaling pathways and molecules associated with metastasis (e.g., epithelial–mesenchymal transition (EMT), tumor microenvironment, extracellular matrix (ECM), genetic and epigenetic factors, cancer stem cells (CSCs), and chronic inflammation).

However, despite the significant anti-metastatic efficacy of carotenoids, as demonstrated especially in preclinical research, specific carotenoids can exhibit rather pro-invasive tendencies (e.g., all-trans retinoic acid) [21,22]. Also, there is an unclear or rather negative association between carotenoids and lung or prostate cancer in specific parts of the population, e.g., smokers [23,24]. Thus, we highlight the anti-cancer effects of carotenoids in the modulation of metastatic processes, as evaluated primarily in preclinical studies. However, we also emphasize the need for an accurate evaluation of the effects of carotenoids in individual types of cancer and the identification of individuals who may benefit from the carotenoid application. Nevertheless, an accurate evaluation of carotenoids’ applicability in metastatic cancer patients, either as individual compounds or in combination with other therapeutics, could lead to the identification of novel agents to improve the treatment outcomes of advanced cancer patients.

## 2. Metastatic Process

**Figure 1:**
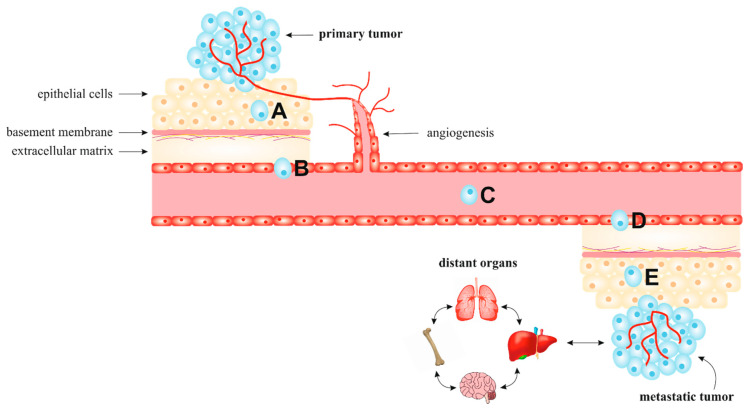
The individual steps of metastasis: local invasion (A), intravasation (B), circulation in blood or lymphatic vessels (C), extravasation (D), and colonization and metastasis (E). Cancer cells from a primary tumor can infiltrate surrounding parenchyma and pass through the blood vessel’s membrane to circulate. The survival rate of circulating tumor cells (CTC) is around 0.2%. Therefore, only a small proportion extravasate and metastasize to target organs as disseminated tumor cells (DTC) [29]. The resulting micrometastatic mass enlarges and colonizes the target organ [30]. Metastatic lesions are primarily detected in specific organ sites (liver, lung, bone, brain) but rarely in others (kidney, heart, stomach) [7].

The metastatic cascade is characterized by disseminating tumor cells from primary tumors to distant tissues to form new tumor colonies [25,26]. As illustrated in Figure 1, metastasis, as a multi-step process, is classified into five different stages: local invasion, intravasation, survival in circulation, extravasation, and colonization and metastasis [27]. Invasion and metastasis, one of the cancer hallmarks, represent advanced and often terminal cancer progression, especially due to current therapies’ incurability. Therefore, about 90% of cancer-associated deaths are caused by metastatic rather than primary tumors [27,28].

### 2.1. Epithelial-Mesenchymal and Mesenchymal-Epithelial Transition

The biological process known as EMT is often involved in physiological (embryogenesis) and pathological (cancer invasion and metastasis) processes. In pathological EMT, polarized epithelial cells lose their adhesive properties and obtain mesenchymal cell phenotypes characterized by increased mobility, such as cell migration, stemness, invasiveness, and drug resistance [31,32]. The loss of E-cadherin expression is considered one of the most important characteristics of EMT. E-cadherin suppression is regulated by transcription factors, including zinc-finger proteins (Snails), E-box-binding proteins (ZEBs), basic helix-loop-helix proteins (Twists), and forkhead box proteins (FOXCs) [33]. Moreover, other transcription factors are involved in the regulation of EMT, such as small non-coding RNAs, epigenetic modulators, and exogenous inducers [34,35,36]. Moreover, the transforming growth factor (TGF), bone morphogenic protein, fibroblast growth factor, epidermal growth factor receptor (EGFR), hepatocyte growth factor (HGF), Wnt/β-catenin, and Notch signaling pathways are involved in EMT regulation [37]. In addition to E-cadherin, the decreased expression of other epithelial markers (zonula occludens-1 (ZO-1) and occludin) is also observed in cancer cells undergoing EMT. However, mesenchymal markers, including N-cadherin, vimentin, fibroblast-specific protein 1, and fibronectin, are increased [38].

Furthermore, the reverse process of EMT, known as mesenchymal-epithelial transition (MET), is associated with stem cell differentiation and de-differentiation. The reprogramming of somatic cells connected to the induction of pluripotent stem cells can allow new opportunities to generate specific cell sources for patients [39,40]. The EMT–MET transition can generate cancer cells associated with increased stemness and the ability to form macro-metastatic colonies [32]. Therefore, targeting EMT pathways provides an attractive strategy for cancer therapy.

### 2.2. Extrinsic Microenvironment and Extracellular Matrix

The initiation of metastasis is not a cell-autonomous event; rather, it is influenced by a complex tissue microenvironment. Cancer metastasizes preferentially to specific organs with a compatible surrounding microenvironments [41]. Similarly, following the Paget hypothesis, proposed at the end of the 19th century, tumor cells can colonize target organs only in favorable microenvironments [42]. Another hypothesis suggested that the surrounding microenvironment influences the cells’ colonization, whereas the spread of tumor cells to distant organs is performed through the circulatory system [43]. In addition to the tumor microenvironment, the ECM controls the regulatory processes strongly affected in malignancy, such as intratumoral signaling, transport mechanisms, metabolism, oxygenation, and immunogenicity.

### 2.3. Regulatory Processes Associated with Metastasis

The maintenance of the ECM is regulated by matrix metalloproteinases (MMPs) and tissue inhibitors of metalloproteinases (TIMPs). During carcinogenesis, the disruption of the balance between MMPs and TIMPs can affect tumor invasiveness and metastasis [44]. The MMP family of zinc-dependent endopeptidases promotes the remodeling of the surrounding environment; this process promotes cell growth and survival through the release of interleukins (IL) and growth factors such as tumor necrosis factor α (TNF-α) and vascular endothelial growth factor (VEGF) [45]. Furthermore, MMPs play a role in cancer migration and ECM turnover by regulating signaling pathways that control cell growth, inflammation, and angiogenesis [46]. MMPs are regulated by urokinase plasminogen activator (uPA), its receptor (uPAR), and TIMPs [47]. Of the four TIMPs, only TIMP-3 binds firmly to the ECM after its secretion [48]. TIMP-3 can bind to many proteinases to inhibit their activity and protect the ECM from degradation; nevertheless, reduced expression of TIMP-3 leads to poor outcomes, including large tumor size, high tumor stage, and metastasis [49]. Moreover, TIMP-1 exerts two different cancer progression roles, likely due to its aberrant glycosylation. On one hand, TIMP-1 interacts with several MMPs and inhibits their matrix-degrading properties [50]. On the other hand, TIMP-1 stimulates cell growth and exhibits anti-apoptotic activity [51,52]. Similarly, except for its MMP-inhibitory activity, TIMP-2 can inhibit cancer progression independently of MMP-mediated mechanisms, probably by modulating tumor cells and the tumor microenvironment [53].

### 2.4. Chronic Inflammation

Inflammation can be a beneficial response with the ability to remove pathogens, repair injured tissue, and restore homeostasis in damaged tissues and organs; however, chronic inflammation has a substantial role in tumor development, including progression, metastasis, and resistance to chemotherapy [46]. Chronic inflammation, one of the cancer hallmarks, leads to changes in epithelial cytoarchitecture and surrounding stromal components associated with the enhancement of genetic/epigenetic aberrations of epithelial cells. This process enhances anti-apoptotic resistance, invasion of adjacent tissues and metastasis to distant organs through the activation of surrounding stromal cells, and the recruitment of immune cells [54]. The expression of TGFβ and IL-10, cytokines derived from tumor cells, is related to the differentiation of tumor-infiltrating immune cells, such as tumor-associated macrophages (TAMs) and neutrophils (TANs), into the tumor-promoting phenotype. The new phenotype of TAMs and TANs can lead to the suppression of anti-tumor immune responses mediated by the production of immunosuppressive cytokines or the expression of T-cell co-inhibitory molecules that can induce mobilization and increase the potential for metastasis [55].

### 2.5. Genetic and Epigenetic Factors

Mutations are typical in tumor cells and can increase oncogenic potential. Chromosomal instability, altered gene expression, and the metastatic process result from morphological adaptations of cancer cells to microenvironmental physical limitations, such as the deformation of the rigid nucleus [56]. Oncogenic mutations might be one of the initial dominant factors that promote metastatic events [57]. For example, a mutation that inactivates the von Hippel-Lindau tumor suppressor leads to the synthesis of hypoxia-inducible factor-1α (HIF-1α). Elevated expression of HIF-1α, especially in renal carcinoma, promotes the colonization of cancer cells via the hyperactivation of a chemokine receptor (CXCR4) in lung parenchyma and bone marrow [58]. Moreover, an activation of EMT can be mediated by epigenetic regulatory processes, including post-translational modifications and also transcriptional factors such as Slug, Snail, and Twist [59]. In cancer, other molecular mechanisms are modified, including the addition of a methyl group that forms 5-methylcytosine at CpG dinucleotides, well characterized DNA cytosine methylation, covalent modification of the histones, and mechanisms involving non-coding RNAs (miRNAs) [60]. The hypermethylation of tumor suppressor (*TP53*, *APC*, and *VHL*) loci is common that is associated with transcriptional silencing [61]. Moreover, miRNAs can act as oncogenes or tumor suppressor with specific function in tumorigenesis, including the control of tumor cell invasion, migration, and metastasis [62,63]. The dysregulation of miRNA expression can lead to altered proliferation, differentiation, apoptosis, development, and metastasis [64].

### 2.6. Cancer Stem Cells (CSCs)

Multilineage differentiation and self-renewal, processes important in both malignant and non-malignant cells, are considered fundamental properties of CSCs [65]. CSCs represent a subpopulation of cells within malignant tumors with specific characteristics, including self-renewal, differentiation, and tumorigenic potential [66]. Moreover, the EMT phenotype stimulates CSC migration, invasiveness, metastasis, and cancer recurrence as well as drug resistance. Subsequently, the MET phenotype allows CSCs to acquire epithelial characteristics in the target tissues [67]. Also, dysregulation of signaling pathways implicated in self-renewal and maintenance of an undifferentiated state of stem cells, such as Notch, Hedgehog, and Wnt, leads to a CSC phenotype [68]. Therefore, the selective targeting of CSCs could be a promising therapeutic strategy against cancer.

## 3. Carotenoids in Cancer Metastasis

Phytochemicals classified as carotenoids, phenolics, alkaloids, nitrogen-containing compounds, and organosulfur compounds possess many beneficial effects in connection to cancer-related metabolic pathways, including cell signaling, cell cycle regulation, cell growth, the oxidative stress response, and inflammation [69,70]. Therefore, plant-derived molecules or whole foods represent potential avenues of cancer chemoprevention and therapy.

**Figure 2:**
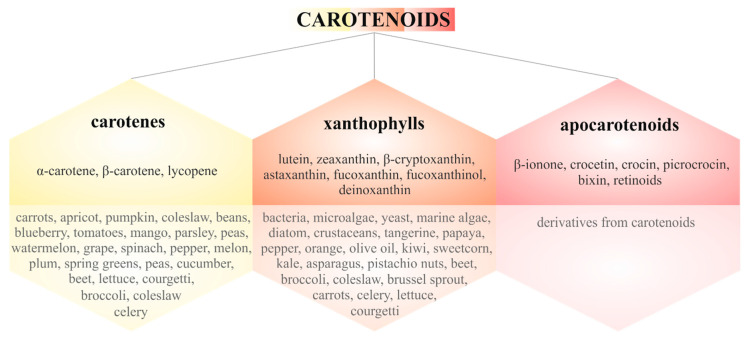
Classification of carotenoids with major representatives and their sources.

Carotenoids are tetraterpenes with a parent hydrocarbon skeleton that consists of C_40_H_56_ with specific alternation in single and double bonds. Furthermore, various cyclic or acyclic end groups of the central carbon chain vary among individual carotenoids. Due to their chemical structure, carotenoids have very low solubility in water [16,71,72]. Moreover, humans cannot synthesize carotenoids, so their intake occurs through the diet or supplementation [73]. Nearly 700 carotenoids are known; these can be classified as carotenes or xanthophylls with their specific color range from yellow to red [74,75,76]. Moreover, carotenoids can be oxidatively cleaved by dioxygenases into carotenoid derivatives known as apocarotenoids [75]. Figure 2 provides an overview of carotenoid classification and the various sources of these natural pigments [77,78,79].

The primary health benefits of carotenoids are based on their antioxidant activity [80]. Moreover, as discussed in our previous review, carotenoids exert positive effects on human health, especially in disease prevention and health maintenance, not only through their antioxidant effects but also through anti-inflammatory effects and enhancement of the immune system [20]. Over the past two decades, carotenoids’ potential roles in carcinogenesis, in both cancer prevention and therapy, were established [81,82]. From a molecular perspective, carotenoids exert anti-cancer effects through the various signaling pathways of proliferation, apoptosis, cell cycle progression, angiogenesis, and metastasis [76]. Moreover, carotenoids can alter the regulatory mechanisms associated with metastasis, including HIF-1α, glucose transporter 1 (GLUT-1), uPA, RhoGTPase (RhoA, Rac1, and Cdc42), MMPs, E-cadherin, surface glycoprotein CD44 and CXCR4, nonmetastatic protein 23 homolog 1 (Nm23-H1), and TIMPs [76]. Due to the high mortality rate caused by metastasis, carotenoids’ anti-tumor effects offer an important strategy to prevent metastasis-associated cancer deaths. Accordingly, various preclinical and clinical studies have focused on the anti-metastatic potential of carotenoids in cancer prevention and therapy.

### 3.1. Carotenoids Modulate Metastatic Processes in Preclinical Research

Carotenoids represent an area of interest in preclinical research targeting metastasis, due to the advanced cancer stages and high patient mortality [27,28]. As discussed below, various in vitro and in vivo cancer studies can serve as an important tool for screening anticancer substances such as carotenoids and elucidating their specific anti-migratory, anti-invasive, and anti-metastatic activities in cancer therapy—these are summarized in Table 1.

#### 3.1.1. Carotenes

Carotenoids can be classified into two groups according to the presence or absence of oxygen in their molecules. Hydrocarbon carotenoids without oxygen in their molecules, such as α-carotene, β-carotene, and lycopene, are collectively called carotenes, which are commonly found in the human diet [20,83].

##### α-Carotene

The anti-metastatic potential of α-carotene was determined in Lewis lung carcinoma in vitro and in vivo. The inhibition of invasion and migration after α-carotene treatment was demonstrated through reduced levels of MMP-2, -9, and uPA, and increased levels of TIMP-1 and plasminogen activator inhibitor (PAI)-1 in murine LLC, BCRC 60,050 Lewis lung carcinoma cells. Moreover, α-carotene inhibited integrin β1-mediated phosphorylation of focal adhesion kinase (FAK) by reducing the phosphorylation of mitogen-activated protein kinase (MAPK). The same results in the inhibition of metastasis without an influence on primary tumor growth were observed in murine C57BL/6 xenografts. In the same study, the enhanced anti-metastatic effect of α-carotene in vivo was determined in a combination with taxol, also known as paclitaxel, an oncologic compound isolated from the bark of the Pacific yew tree (*Taxus brevifolia*) [84].

##### β-Carotene

β-carotene exerted protective effects against tobacco smoke-induced gastric cancer in BALB/c mouse smoking model. The anti-metastatic and chemopreventive potential of β-carotene was underscored by the inhibition of the Notch pathway and EMT alterations, especially increased levels of epithelial markers (E-cadherin, ZO-1, and cytokeratin 5 (CK5)) and reduced expression of mesenchymal markers (Snail-1, vimentin, and N-cadherin) [85]. Furthermore, β-carotene inhibited the invasion and metastasis of neuroblastoma in vitro and in vivo. The reduced migratory and invasive capabilities of SK-N-BE(2)C neuroblastoma cells after β-carotene treatment were demonstrated by the suppression of MMP-2 under both normoxic and hypoxic conditions. Moreover, in metastasis-induced SK-N-BE(2)C nude mice, the administration of β-carotene reduced tumor volume and liver metastasis associated with lower levels of MMP-2, -9, and membrane-type (MT) 2 MMP, when compared to controls. TIMP-1 and -2 levels also decreased, suggesting their independent association with MMPs. The potential anti-metastatic action of β-carotene was attributed to the inhibition of HIF-1α and its downstream target genes, specifically the angiogenic factor VEGF and the glucose transporter GLUT1 under hypoxic conditions [86]. Moreover, β-carotene-treated M2 macrophages and activated fibroblasts, both playing a key role in the behavior modulation of cancer cells in the tumor microenvironment, inhibited CSC markers (CD133, CD44, SOX2, and NOTCH1), and modulated EMT markers (increased E-cadherin) and the IL-6/STAT3 signaling pathway. They thereby decreased colon cancer cell invasiveness and migration in HCT116 cells and an azoxymethane/dextran sodium sulfate-induced colitis-associated colorectal cancer model in male BALB/c mice. The data suggest that the anti-cancer activity of β-carotene is related to the inhibition of M2 macrophage polarization and fibroblast activation (alpha-smooth muscle actin (α-SMA), fibroblast activation protein alpha (FAP), and TGF-β1) [87]. Interestingly, the enzyme β-carotene 15,15′-oxygenase (BCO1) catalyzes the first step of the biosynthesis of vitamin A, which is important for neuroblastoma differentiation. BCO1 inhibited self-renewal and neuroblastoma CSC markers (DLK1, NOTCH1, SOX2, CD44, and CD133) in BE(2)C cells. The metastatic potential of BE(2)C cells was suppressed by decreased levels of MMP-2, -9, and HIF-1α and its downstream targets VEGF and GLUT1. Expression of MT MMPs and TIMPs can trigger tumor spread by activating gelatinase A. In this study, the expression of MT1-MMP, MT2-MMP, TIMP-1, and -2 decreased. Moreover, the overexpression of BCO1 could revert the EMT progression by increasing E-cadherin and decreasing N-cadherin and vimentin levels. Similar effects were detected in murine BE(2)C xenografts [88].

##### Lycopene

Lycopene, a bright red carotene pigment, showed anti-metastatic effects in SK-Hep-1 liver adenocarcinoma cells through the inhibition of the expression of NADPH oxidase 4 (NOX4), a protein with a pivotal role in the production of reactive oxygen species (ROS). In TGF-β-induced metastasis, lycopene administration inhibited migration, invasion, and adhesion, as well as MMP-9, -2, and ROS levels, suggesting that down-regulation of NOX4 plays a critical role in the anti-metastatic action of lycopene in vitro [89]. Moreover, lycopene inhibited the EMT in murine CAL-27 oral cancer xenografts, as demonstrated by higher E-cadherin levels and reduced N-cadherin levels. Furthermore, lycopene inhibited the in vitro migration of CAL-27 and SCC-9 oral cancer cells [90]. The anti-metastatic effect of lycopene was also observed through the down-regulated expression of *ITGA5* and *ITGB1* [91], key players in ovarian cancer cell invasion and metastasis [92], *MMP-9*, and EMT markers (TWIST, ZEB2, SNAI-1 and -2, FOXC2, FN1, TGFB-1 and -2, TGFBR1, and SMAD4) in OV-MZ-6 ovarian cancer cells [91]. Moreover, apo-10′-lycopenoic acid (ALA), a derivative of lycopene, induced peroxisome proliferator-activated receptor gamma (PPARγ) activation and consequent angiogenic inhibition, which ultimately prevented the migration and metastasis of HuH7 liver and A549 lung cancer cells through the inhibition of MMP-2 [93]. Impressively, lycopene-enriched tomato extract (LETE) reduced different markers of hypoxia, angiogenesis, and metastasis, including HIF-1α, VEGF, CD31, MMP-2, and -9, in an initial stage of N-nitrosodiethylamine (NDEA)-induced hepatocellular carcinoma of female BALB/c mice. These results reflect the association of these markers with cancer progression, and LETE’s potential role as a strategy in cancer treatment [94].

#### 3.1.2. Xanthophylls

Xanthophylls are the naturally occurring oxygenated derivatives of hydrocarbon carotenoids, commonly synthesized by plants and microorganisms. Major xanthophylls in the human diet, including lutein, zeaxanthin, and β-cryptoxanthin, and xanthophylls found in bacteria, yeast, and algae, including astaxanthin and fucoxanthin, exert anti-cancer properties [20,95].

##### Astaxanthin

Astaxanthin (AST), a carotenoid commonly found in plants and seafood, increased the expression of miR-29a-3p and miR-200a, leading to the suppression of MMP-2 and ZEB1, as well as the EMT, in human HCT116 and murine CT26 colorectal cancer cells. In addition, the mouse colon cancer model revealed that AST’s anti-metastatic activity is related to the repression of the transcription factor MYC [96]. Moreover, Siangcham et al. (2020) demonstrated metastastis-reducing effects of AST through the suppression of MMP-2 and -9 in A172 human glioblastoma cells [97]. Interestingly, the migration assay demonstrated an immediate decrease in the migration of two MCF-7 ER^+^ and MDA-MB-231 breast cancer cell lines immediately after AST treatment [98]. Similarly, AST combined with human serum albumin inhibited the migration of SKOV3 ovarian cancer cells [99]. The precise inhibitory mechanisms involved in both of these cases require further elucidation. Impressively, the probiotic yeast *Kluveromyces marxianus*, which produces configurational stereoisomers of AST (3S, 3′S), inhibited the lung metastasis of B16F10-PKH26 murine melanoma cells. On the other hand, strong invasion, especially of the thorax and lungs, occurred in the melanoma group without AST [100].

##### Fucoxanthin

Fucoxanthin, a major active component extracted from *Laminaria japonica*, inhibited the in vitro migration and invasion of A549, H1299, and H446 lung cancer cells. This effect correlated with the reduced expression of Snail, Twist, fibronectin, N-cadherin, and MMP-2, inhibition of the PI3K/AKT/NF-κB pathway, and increased expression of TIMP-2. Moreover, the observed anti-metastatic effect was consistent with murine PC9 xenografts [101]. In another study, a subtoxic dose (5 µM) of fucoxanthin demonstrated anti-invasive and anti-metastatic potential by decreasing the levels of stemness-related proteins (Wnt-1 and β-catenin), EMT markers (fibronectin, MMP-2, and vimentin), and an angiogenic factor (VEGF) in p53 wild-type U2OS osteosarcoma and p53 null SKOV3 ovarian cancer cells [102]. Furthermore, fucoxanthin treatment suppressed MMP-2, -9, and uPA by reducing the phosphorylation of p38 in U87 and U251 human glioblastoma cells. The anti-migratory and anti-invasive effects of fucoxanthin are attributed to its ability to control the translocation of U87 cells to adjacent tissue or distant organs [103].

Additionally, a fucoxanthin metabolite known as fucoxanthinol (FxOH) exhibits anti-cancer and anti-metastatic effects similar to fucoxanthin. FxOH reduced EMT by inhibiting N-cadherin, vimentin, and activating integrin signaling in colorectal CSCs. Moreover, in a dose-dependent manner, FxOH inhibited sphere formation. These effects were associated with suppressed migration and invasion [104].

##### Other Xanthophylls

β-cryptoxanthin inhibited the migration of AGS and SGC-7901 gastric cancer cells, as demonstrated by decreased protein levels of MMP-2 and -9 and suppressed tumor growth in murine AGS xenografts. Moreover, the authors concluded that the anti-metastatic effect of β-cryptoxanthin was connected to reduced VEGF and epidermal growth factor (EGF), and enhanced apoptosis mediated by the inactivation of AMP-activated protein kinase (AMPK) signaling [105].

Moreover, in MDA-MB-157 and MCF-7 breast cancer cells under hydrogen peroxide-induced hypoxic conditions, lutein treatment modulated the expression of EMT-associated factors (increased E-cadherin and decreased vimentin and N-cadherin) and reduced NOTCH signaling associated with the inhibition of tumor invasion and migration. Moreover, HIF-1α and hairy and enhancer of split 1 (HES1), which are associated with hypoxia-induced invasion and EMT activation, were downregulated after lutein treatment. These results were associated with the presence of the ROS scavenger N-acetylcysteine, which reduced ROS levels in both cell lines under hydrogen peroxide-induced hypoxia [106].

The xanthophyll zeaxanthin also inhibited the migration and invasion of C918 cultured uveal melanoma cells by downregulating MMP-2. Interestingly, decreased levels of NF-*κ*B, an affector upstream of MMP-2 secretion, were observed in the nuclear extracts of C918 cells, suggesting that its modulation could support the prevention of metastasis [107].

#### 3.1.3. Apocarotenoids

Apocarotenoids have shortened carbon skeletons as a result of oxidative cleavage. These carotenoids include retinoids, vitamin A, β-ionone, α-ionone, bixin, crocin, and crocetin [20,83].

##### All-Trans Retinoic Acids

All-trans retinoic acids (ATRA), the active derivatives of vitamin A, inhibited metastasis by reducing M2 polarization of TAMs in mice injected with K7M2 WT osteosarcoma cells. ATRA also decreased the metastatic pulmonary nodes of osteosarcoma. Moreover, ATRA reduced IL13-induced secretion of MMP-12 [108]. Similarly, ATRA treatment inhibited tumor nodules in C57BL/6 mice injected with B16F10 murine melanoma cells compared to metastatic controls, especially in the lungs and liver [109]. Furthermore, a long period of paclitaxel administration leads to the EMT phenotype and related metastasis and resistance. However, ATRA treatment reversed the EMT by inhibiting NF-κΒ and upregulating gap junctions in the paclitaxel-resistant HCT116, LoVo, and CT26 colorectal cancer cell lines. Moreover, EMT marker expression was altered after ATRA treatment: fibronectin, MMP-9, N-cadherin, Snail, vimentin, and β-catenin levels decreased, while E-cadherin levels increased. ATRA’s anti-metastatic effect, via a decreased number of cancer nodules, was also observed in BALB/c mice injected with CT26 murine colon cancer cells [110]. Another study that focused on the anti-invasive and anti-metastatic potential of ATRA in colon cancer revealed cell movement inhibition and increased cell adhesion in human RKO cell lines. Myosin light chain kinase (MLCK), commonly phosphorylated and activated by MAPK, decreased after ATRA treatment. ATRA also increased occludin and ZO-1 expression on the RKO cell membrane. In fact, a MAPK inhibitor (PD98059) enhanced ATRA’s inhibitory effect on RKO migration. In addition, knockdown of the extracellular signal-regulated kinase (ERK) reduced MLCK expression; this suggests that migration of colon cancer cells could be diminished by ATRA treatment and the consequent modulation of the ERK1/MAPK signaling pathway [111]. Furthermore, ATRA inhibited the colony formation, migration, and invasion of mouse hepa1-6 hepatocarcinoma cells by modulating EMT markers. Treatment with ATRA reduced the mesenchymal markers N-cadherin, vimentin, Snail, and Twist, but increased the epithelial marker E-cadherin, in Hepa1-6 cells [112].

Paradoxically, despite the demonstrated anti-metastatic properties of ATRA in specific cancer types, ATRA can promote cancer growth and invasion in other cancer lines. As demonstrated by Mezquita et al. (2018), ATRA exerted pro-invasive effects through the activation of the Src-YAP-IL6 axis in MDA-MB-231 breast cancer cells. On the other hand, ATRA inhibited Src-YAP-IL6 in MDA-MB-468 cells, resulting in the mitigation of the invasion phenotype. The inhibition of the Src-YAP-IL6 axis in both cell lines leads to reduced migration and invasion [21]. A similar pro-metastatic effect was observed in THP-1 human myeloid leukemia cells in which ATRA induced MMP-2 expression and secretion in a calcium ion-dependent manner, via the retinoic acid receptor (RAR) and retinoid X receptor (RXR) signaling pathways [22].

##### Crocin and Crocetin

The main two natural carotenoids of saffron (*Crocus sativus* L.), crocetin and its digentiobiosyl ester crocin, have anti-metastatic potential in different cancer types. In 4T1 mammary carcinoma cells with highly invasive and metastatic characteristics, crocin and crocetin inhibited migration, cell mobility, invasion, and attenuated adhesion to the extracellular matrix. In the evaluation of the mRNA expression of Wnt/β-catenin target genes including Frizzled-7 (*FZD7*), developmentally downregulated protein 9 (*NEDD9*), and *VEGF-α*, crocin revealed a stronger effect on their down-regulation when compared to crocetin treatment as well as the control group; however, both crocin and crocetin reduced vimentin (*VIM*) expression. Also, upregulation of E-cadherin (*E-CAD*) was observed after crocin treatment [113]. Moreover, a detailed study of crocin and crocetin’s combination revealed their anti-metastatic effects through the reduction of metastatic foci in livers and lungs in a murine triple-negative breast cancer model [114]. In another study, crocin reduced metastasis in BALB/c mice injected with 4T1 cells by inhibiting the expression of Wnt/β-catenin target genes (*NEDD9*, *VEGF-α*, *MMP-9*, *FZD7*, and *VIM*), especially in metastatic tissues such as the liver and lungs [115]. Furthermore, crocin inhibited the migration and invasion of AGS and HGC-27 gastric cancer cells by a reduced level of Krüppel-like factor 5 (KLF5), HIF-1α, and EMT activity by increasing E-cadherin and decreasing Snail and N-cadherin. Moreover, reduced expression of KLF5 after crocin treatment was associated with increased levels of miR-320. Although the relationship between KLF5 and HIF-1α in gastric cancer is not well defined, KLF5 is a known transactivator of HIF-1α in colon cancer [116]. Additionally, Janus kinase (JAK) and signal transducer and activator of transcription (STAT) are signals normally involved in cancer pathologies such as cell differentiation, migration, and proliferation [117]. Crocin inhibited STAT3 activation through the inactivation of JAK1, JAK2, and Src kinase in IL-6-stimulated Hep3B and HepG2 liver cancer cells. Crocin also decreased CXCR4 and VEGF protein levels, pointing to its anti-invasive potential in both liver cancer cell lines [118].

##### Other Apocarotenoids

Several potential candidates as novel anticancer therapeutics against various cancer types and associated metastasis have been published. The administration of VNLG-152, a novel retinamide, modulated EMT activity in 22Rv1 prostate cancer cells by increasing E-cadherin expression and decreasing N-cadherin, β-catenin, MMP-2, -9, claudin, vimentin, Snail, Slug, and Twist expression [119]. Moreover, alkylamide derivatives of bexarotene, DK-1–150 and DK-1–166, inhibited migration, modulated CSC markers (c-Myc, KLF4, Nanog, Oct4A, and SOX2), and EMT activities (increased E-cadherin) of BT549 and MDA-MB-231 triple-negative breast cancer cell lines [120]. Furthermore, N-(4-hydroxyphenyl)retinamide, also known as fenretinide is a synthetic analog of ATRA that inhibited HepG2 liver cancer cell migration by increasing E-cadherin expression and p38-MAPK phosphorylation, by reducing MLCK activation [121].

Despite the beneficial effects of carotenoids against metastasis in various cancer types, the use of carotenoids in some cases can lead to pro-invasive behavior, as demonstrated by ATRA use in cancer treatment [21,22]. Therefore, a more precise evaluation of carotenoid efficacy and the determination of detailed mechanisms can support a better understanding of carotenoids’ anti-cancer properties, especially in cancer prevention and therapy, for safe, efficient, and timely clinical trials.

### 3.2. Nanoparticles Conjugated with Carotenoids as a Novel Strategy in Cancer Management

Nowadays, nanotechnology has opened up new treatment options in cancer management. Targeting tumor cells with nanoparticles (NPs) seems to be promising in the prevention of metastatic spread [122]. Due to low bioavailability and solubility of carotenoids, nanotechnology can significantly influence their use, especially for specific drug delivery to cancer sites [123]. Moreover, NPs can reduce or eliminate the adverse effects of anti-cancer drugs [124].

Co-delivery of ATRA and paclitaxel using human serum albumin-bound (ATRA/PTX/HSA) NPs notably reduced MMP-2 and -9 levels in highly metastatic 4T1 mouse breast cancer cells and a murine 4T1 breast tumor model when compared with PTX-NPs or HSA-NPs loaded with single drugs [125]. Similarly, a combination of PTX/ATRA-incorporated NPs decreased the activity of MMP-2 and consequently inhibited the invasion of CT26 colon carcinoma cells [126]. Interestingly, magnetite NPs coated with crocin suppressed precancerous lesions, markers of cell proliferation, inflammation, oxidative stress, and angiogenesis, leading to the prevention of metastasis in HepG2 liver cancer cells and diethylnitrosamine-injected mice [127]. Moreover, the nanoemulsion system incorporating the lycopene and gold NPs demonstrated anti-migration activity in HT-29 colon cancer cells by downregulating the expression of Akt, NF-κB, MMP-2, and -9, and upregulating the expression of the epithelial marker E-cadherin [128]. Notably, gastric CSCs, especially CD44 and CD133 markers, are commonly responsible for the initiation, recurrence, metastatic spread, and drug resistance of gastric cancer [129,130]. The innovation of CD44 and CD133 antibody-conjugated ATRA-loaded poly (lactide-co-glycolide)-lecithin-PEG NPs could specifically target CD44^+^ and CD133^+^ gastric CSCs, leading to the inhibition of tumor growth. It was first mentioned that CD44 and CD133 antibodies could fight against cancer progression [131].

Encapsulation of carotenoids in different nanocarriers (polymeric/biopolymeric, lipid-based, inorganic, and hybrid nanocarriers) could represent an innovative strategy for improving human health because of their enhancement of solubility, cellular uptake, membrane permeation, bioaccessibility, and stability [132,133]. Especially, lipid-based nano-delivery cargos, including nano-liposomal vehicles, solid lipid nanoparticles, nano-emulsions, and nano-structured lipid carriers, could increase the solubility and bioavailability of carotenoids and control their release in pharmaceutical or food applications [134,135]. As was mentioned above, carotenoids in nanocarriers and conjugated with other drugs/substances can effectively reduce cancer progression and metastasis and represent a potential tool in cancer management.

### 3.3. Carotenoids and Their Anti-Metastatic Effects in Clinical Practice

Various carotenoids, e.g., synthetic retinoid, bexarotene, and/or ATRA, have anti-cancer properties and can thus be used for maintenance therapy, such as for improvement of the immune system by increasing the levels of lymphocyte and natural killer (NK) cells [136,137], or for disease stabilization of advanced hepatocellular carcinoma, metastatic melanoma, and recurrent or metastatic melanoma, respectively [138,139,140]. NK cells can exert robust anti-metastatic functions by killing damaged, infected, or (pre)malignant cells [141]. Moreover, supplements with lycopene and/or plasma carotenoids also have potential in advanced prostate and bladder cancer prevention [142,143]. Similarly, combinations of carotenoids with anti-cancer drugs or other natural substances, e.g., tamoxifen with retinyl acetate and/or ATRA combined with oxaliplatin and 5-fluorouracil/leucovorin (FOLFOX), exhibit synergistic effects in breast cancer and advanced hepatocellular carcinoma with extrahepatic metastasis, respectively [144,145]. With respect to whether carotenoids can modulate metastatic processes, there is a lack of evidence of more accurate mechanisms of carotenoids against tumor progression and metastatic spread.

Anti-metastatic therapies in differentiated thyroid carcinoma (DTC) patients are limited due to decreased radioiodide uptake (I-131). Bexarotene treatment partially restored I-131 uptake in the majority of patients (8/11). However, the uptake was detected only at single-photon emission tomography (SPECT) but not present in all metastases visualized by computed tomography (CT) scanning, likely due to heterogeneity of DTC [146]. Moreover, a study protocol for a forthcoming randomized controlled trial (ID: ChiCTR-IIR-17012916) assumes that ATRA combined with FOLFOX could inhibit advanced hepatocellular carcinoma and associated extrahepatic metastasis of 368 patients [145]. On the contrary, not all clinical studies met the expectations that carotenoids exert beneficial effects against cancer progression and metastasis. Some treatment combinations should not be recommended for cancer patients, as was demonstrated in patients with metastatic renal cell carcinoma. ATRA in combination with interferon-α did not improve the response of interferon in these patients [147].

**Figure 3:**
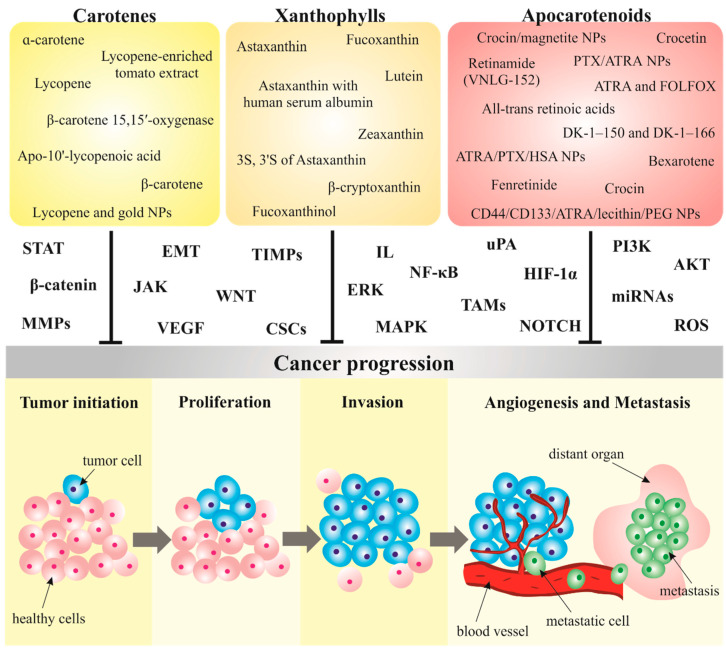
Carotenoids targeting metastasis in preclinical and clinical cancer research. Abbreviations: MMPs, matrix metalloproteinases; CSCs, cancer stem cells; STAT, Signal transducer and activator of transcription; ROS, reactive oxygen species; uPA, urokinase plasminogen activator; TIMPs, tissue inhibitors of metalloproteinases; MAPK, mitogen-activated protein kinase; HIF-1α, hypoxia inducible factor-1α; VEGF, vascular endothelial growth factor; IL, interleukin; EMT, epithelial-mesenchymal transition; PI3K, phosphoinositide 3-kinases; AKT, protein kinase B; NF-κB, nuclear factor-κB; TAMs, tumor-associated macrophages; ERK, extracellular signal-regulated kinase; JAK, Janus 447 kinase.

The effects of carotenoids on metastatic pathways in clinical trials need to be further analyzed due to the diverse outcomes across different cancer types. It is important to evaluate more accurate results that define anti-cancer effects and carotenoids’ mechanisms or their role in cancer prevention. Figure 3 summarizes preclinical and clinical research focused on the anti-metastatic properties of carotenoids.

#### Limitations of Carotenoids

Clinical studies are commonly limited by side effects of treatment, toxicity, bioavailability, and/or safety. Carotenoids’ low bioavailability and solubility (as a result of their lipophilic natures) also limit their pharmacological use [132].

Various studies revealed temporary toxicity of carotenoids; however, the cessation of use or reduced dosage during treatment can abolish side effects. Patients with DTC tolerated treatment with bexarotene well. However, in three patients, the dose was reduced because of hypertriglyceridemia and leukopenia [146]. Moreover, bexarotene induced grade 3 myalgia, asthenia, diarrhea, cold hands/feet, and mood changes, but myelosuppression was mild in patients with metastatic melanoma [139]. The most common side effects (more than 50%) of bexarotene in a study of 16 Japanese adults with T-cell lymphoma included hypothyroidism, hypertriglyceridemia, hypercholesterolemia, leukopenia, and neutropenia [148]. Furthermore, therapy with fenretinide demonstrated reversible mucotaneous toxicities in 52% of tested breast and melanoma cancer patients but returned to baseline after 2–4 weeks of treatment or after reduced dosages [149]. Fenretinide also induced reversible toxicity, including visual changes (haziness, altered night vision), nausea/vomiting, and diarrhea in the pathogenesis of small cell lung cancer [150]. In contrast, treatment with lycopene showed mild toxicity in 36 patients with recurrent prostate cancer after definitive local therapy. Only one patient terminated therapy prematurely due to diarrhea [151]. Similarly, dietary lycopene intake (tomato juice) was well tolerated without any gastrointestinal side effects in 20 men with prostate cancer undergoing intensity-modulated radiation therapy [152]. In the same way, lycopene appears to be effective, safe, and relatively innocuous in treating hormone-refractory metastatic prostate cancer and should be used in therapy before more toxic substances [153]. It is not surprising that lycopene also has protective effects against natural (mycotoxins and bacterial toxins) and chemical (heavy metals, pesticides, and herbicides) toxic substances, whose high concentrations can lead to carcinogenesis [154].

Poor absorption and bioavailability represent the main disadvantages of carotenoids [155]. Their low water solubility is related to their glycosylated form [156]. Several methods can positively influence bioavailability and solubility, including specific cooking methods, biofortification, solid dispersion, microemulsions [20], as well as earlier mentioned lipid-based nano-delivery cargos [134,135]. Moreover, the determination of carotenoids’ content in different food forms (juice, raw, cooked) may contribute to the variability in serum carotenoid responses to vegetable and fruit interventions in clinical studies. For example, the serum levels of α-carotene and lutein increased after vegetable juice uptake compared to raw or cooked vegetable [157]. Additionally, patient stratification plays an important role in cancer prevention. As demonstrated in a lung cancer chemoprevention study, smoking and alcohol consumption by men and women with a documented history of occupational asbestos exposure led to lower concentrations of serum β-carotene, even after adjustment of carotenoid intake. Also, retinol levels decreased by smoking and increased by alcohol consumption [158]. However, recent studies have demonstrated pro-cancerous effects of β-carotene, especially in smokers. High doses of β-carotene failed to exhibit chemopreventive activities in several clinical trials [159]. For example, β-carotene increased the risk of lung cancer in smokers [23], and high concentrations of serum retinol and α-carotene increased the risk of total and high-grade prostate cancers [24]. Moreover, it is necessary to consider intraindividual characteristics, such as lifestyle, diet, physiologic factors, or other diseases, which affect the effectivity of carotenoids [160]. The common adverse effect of carotenoid intake, including retinoids or bexarotene, in cancer treatment are related to hypercholesterolemia [148,161]. Thus, we highlight the importance of patient stratification, e.g., patients with high cholesterol, to consider all effects of carotenoids in cancer therapy or prevention in a specific population. Therefore, it is important to evaluate more precise mechanisms of potential anti-cancer agents or supplements such as carotenoids, especially for the safety of patients and the prevention of adverse effects [162,163].

## 4. Conclusions and Outlook

All data mentioned in this review strongly suggest that different groups of carotenoids may target multiple molecular signaling pathways associated with metastatic cancer. Several recent studies described the synergistic anti-metastatic effects of carotenoids combined with other molecules applied in standard cancer therapies. However, in-depth mechanistic preclinical and clinical studies need to be conducted to validate the reliable anti-cancer and anti-metastatic efficacy of carotenoids. With regard to improved cellular uptake, membrane transport, bioaccessibility, and stability of carotenoids, a very prospective strategy in cancer management seems to be the application of carotenoids in nanocarriers or nanoparticles conjugated with other effective (conventional) cancer drugs. This approach represents a way for a more effective clinical application of carotenoids in advanced cancer disease. Nevertheless, it is important to define effective and safe doses of carotenoids in humans. To this end, epidemiological studies indicate that excessive amounts of certain carotenoids may exert adverse side effects in organisms. Based on the preclinical and clinical reports reviewed in this paper, we can conclude that carotenoids represent prospective candidate molecules for oncology research in metastatic cancer, with a high potential for successful future applications in clinical practice.

How to proceed practically? The application of carotenoids in metastatic disease management of different origin is considered as a potentially effective mitigating measure, stratifying patients by individual diagnosis, dosing, and safety of medication [20,114,164]. At the level of primary prevention, innovative screening programs are essential to identify persons at high risk to develop particularly aggressive cancer sub/types who might be strongly predisposed to metastatic disease [164,165,166,167,168,169]. At the level of secondary prevention, cancer patients should undergo predictive diagnostics estimating individual metastatic potential. Recently prominent examples have been presented in the literature [164,170,171]. Various carotenoids, including β-carotene, lycopene, and retinoids and their receptors, can effectively fight against premalignant lesions in the role of secondary cancer prevention [172,173,174,175]. In palliative care of metastatic disease, optimized management has been proposed based on the medical application of artificial intelligence such as an unsupervised machine learning which considers a large spectrum of parameters, treatment algorithms, and mitigating measures tailored to the person, such as optimal therapy approaches [20,176].

## Figures and Tables

**Table 1:** Carotenoids targeting migration, invasion, and metastasis in preclinical cancer research.

Carotenoids Group	Carotenoids	Study Design	Mechanism	Ref.
Carotenes	α-carotene	Murine LLC, BCRC 60,050 Lewis lung carcinoma cells; murine C57BL/6 xenografts	↓ MMP-2, ↓ MMP-9, ↓ uPA, ↑ TIMP-1, ↑ PAI-1, ↓ integrin β1-mediated phosphorylation of FAK, ↓ MAPK	[84]
	β-carotene	BALB/c mouse smoking model	↓ Notch pathway, ↑ E-cadherin, ↑ ZO-1, ↑ CK5, ↓ Snail-1, ↓ vimentin, ↓ N-cadherin	[85]
		SK-N-BE(2)C neuroblastoma cells; SK-N-BE(2)C nude mice	↓ MMP-2, ↓ MMP-9, ↓ MT2 MMP, ↓ TIMP-1, ↓ TIMP-2, ↓ HIF-1α, ↓ VEGF, ↓ GLUT1	[86]
		HCT116 colorectal cancer cells; β-carotene-treated M2 macrophages and activated fibroblasts of azoxymethane/dextran sodium sulfate-induced colitis-associated colorectal cancer of male BALB/c mice	↓ CSC markers (CD133, CD44, SOX2, and NOTCH1), ↓ invasiveness, ↓ migration, ↑ E-cadherin, ↓ IL-6/STAT3 signaling pathway, ↓ M2 macrophage polarization, ↓ fibroblast activation (α-SMA, FAP, and TGF-β1)	[87]
	β-carotene 15,15′-oxygenase	BE(2)C neuroblastoma cells; murine BE(2)C xenografts	↓ self-renewal, ↓ CSCs markers (DLK1, NOTCH1, SOX2, CD44, and CD133), ↓ MMP-2, ↓ MMP-9, ↓ MT1-MMP, ↓ MT2-MMP), ↓ TIMP-1, ↓ TIMP-2, ↓ HIF-1α, ↓ VEGF, ↓GLUT1, ↑ E-cadherin, ↓ N-cadherin, ↓ vimentin	[88]
	Lycopene	SK-Hep-1 liver adenocarcinoma cells	↓ NOX4, ↓ ROS, ↓ MMP-9, ↓ MMP-2	[89]
		CAL-27 and SCC-9 oral cancer cells; murine CAL-27 oral cancer xenografts	↑ E-cadherin, ↓ N-cadherin, ↓ migration	[90]
		OV-MZ-6 ovarian cancer cells	↓ *ITGA5*, ↓ *ITGB1*, ↓ *MMP-9*, ↓ EMT markers (TWIST, ZEB2, SNAI-1 and -2, FOXC2, FN1, TGFB-1 and -2, TGFBR1, and SMAD4)	[91]
	Lycopene-enriched tomato extract	N-nitrosodiethylamine (NDEA)-induced hepatocellular carcinoma of female BALB/c mice	↓ HIF-1α, ↓ VEGF, ↓ CD31, ↓ MMP-2, ↓ MMP-9	[94]
	Apo-10′-lycopenoic acid	HuH7 liver and A549 lung cancer cells	↑ PPARγ, ↓ MMP-2	[93]
Xanthophylls	Astaxanthin	Human HCT116 and murine CT26 colorectal cancer cells; murine colon cancer model	↑ miR-29a-3p, ↑ miR-200a, ↓ MMP-2, ↓ ZEB1, ↓ EMT, ↓ MYC	[96]
		A172 human glioblastoma cells	↓ MMP-2, ↓ MMP-9	[97]
		MCF-7 ER^+^ and MDA-MB-231 breast cancer cells	↓ migration	[98]
	Astaxanthin with human serum albumin	SKOV3 ovarian cancer cells	↓ migration	[99]
	configurational stereoisomers 3S, 3′S of AST	Mice injected with B16F10-PKH26 mouse melanoma cells	↓ lung metastasis	[100]
	Fucoxanthin	A549, H1299, H446 lung cancer cells; murine PC9 xenografts	↓ Snail, ↓ Twist, ↓ fibronectin, ↓ N-cadherin, ↓ MMP-2, ↓ PI3K/AKT/NF-κB pathway, ↑ TIMP-2	[101]
		p53 wild-type U2OS osteosarcoma and p53 null SKOV3 ovarian cancer cells	↓ Wnt-1, ↓ β-catenin., ↓ fibronectin, ↓ MMP-2, ↓ vimentin, ↓ VEGF	[102]
		U87 and U251 human glioblastoma cells	↓ MMP-2, ↓ MMP-9, ↓ uPA, ↓ phosphorylation of p38	[103]
	Fucoxanthinol	Colorectal CSCs	↓ N-cadherin, ↓ vimentin, ↑ integrin signaling, ↓ sphere-formation, ↓ migration, ↓ invasion	[104]
	β-cryptoxanthin	AGS and SGC-7901 gastric cancer cells; murine AGS xenografts	↓ MMP-2, ↓ MMP-9, ↓ VEGF, ↓ AMPK signaling, ↑ apoptosis	[105]
	Lutein	MDA-MB-157 and MCF-7 breast cancer cells	↑ E-cadherin, ↓ vimentin, ↓ N-cadherin, ↓ NOTCH signaling, ↓ invasion, ↓ migration, ↓ HES1, ↓ ROS, ↑ hydrogen peroxide	[106]
	Zeaxanthin	C918 cultured uveal melanoma cells	↓ MMP-2, ↓ NF-*κ*B, ↓ migration, ↓ invasion	[107]
Apocarotenoids	All-trans retinoic acids	Mice injected with K7M2 WT osteosarcoma cells	↓ M2 polarization of TAMs, ↓ pulmonary metastatic nodes of osteosarcoma, ↓ MMP-12	[108]
		C57BL/6 mice injected with B16F10 murine melanoma cells	↓ tumor nodules in lungs and liver	[109]
		Paclitaxel-resistant HCT116, LoVo and CT26 colorectal cancer cells; BALB/c mice injected with CT26 murine colon cancer cells	↓ NF-κΒ, ↑ gap junctions, ↓ fibronectin, ↓ MMP-9, ↓ N-cadherin, ↓ Snail, ↓ vimentin, ↓ β-catenin, ↑ E-cadherin	[110]
		RKO human colon adenocarcinoma cells	↓ cell movement, ↑ cell adhesion, ↓ MLCK, ↑ occludin, ↑ ZO-1, ↓ ERK1/MAPK signaling pathway	[111]
		Murine hepa1-6 hepatocarcinoma cells	↓ colony formation, ↓ migration, ↓ invasion, ↓ N-cadherin, ↓ vimentin, ↓ Snail, ↓ Twist, ↑ E-cadherin	[112]
	Crocin and crocetin	4T1 mammary carcinoma cells	↓ migration, ↓ cell mobility, ↓ invasion, ↓ adhesion to extracellular matrix, ↓ Wnt/β-catenin, ↓ *FZD7*, ↓ *NEDD9*, ↓ *VEGF-ɑ*, ↓ vimentin, ↑ E-cadherin	[113]
		Murine triple negative breast cancer model	↓ metastatic foci in livers and lungs	[114]
	Crocin	BALB/c mice injected with 4T1 mammary carcinoma cells	↓ Wnt/β-catenin target genes (*NEDD9*, *VEGF-ɑ*, *MMP-9*, *FZD7* and *VIM*)	[115]
		AGS and HGC-27 gastric cancer cells	↓ KLF5, ↓ HIF-1α, ↑ miR-320, ↓ migration, ↓ invasion, ↑ E-cadherin, ↓ Snail, ↓ N-cadherin	[116]
		IL-6-stimulated Hep3B and HepG2 liver cancer cells	↓ STAT3, ↓ JAK1, JAK2, ↓ Src kinase, ↓ CXCR4, ↓ VEGF, ↓ invasion	[118]
	Retinamide (VNLG-152)	22Rv1 prostate cancer cells	↑ E-cadherin, ↓ N-cadherin, ↓ β-catenin, ↓ MMP-2, ↓ MMP-9, ↓ claudin, ↓ vimentin, ↓ Snail, ↓ Slug, ↓ Twist	[119]
	Alkylamide derivatives of bexarotene DK-1–150 and DK-1–166	BT549, and MDA-MB-231 triple-negative breast cancer cell lines	↓ migration, modulated CSC markers (c-Myc, KLF4, Nanog, Oct4A, and SOX2), ↑ E-cadherin	[120]
	Fenretinide	HepG2 liver cancer cells	↓ migration, ↑ E-cadherin, ↑ phosphorylation of p38-MAPK, ↓ MLCK	[121]

Explanatory notes: ↑ increased/induced; ↓ decreased/inhibited. Abbreviation: MMPs, matrix metalloproteinases; uPA, urokinase plasminogen activator; TIMPs, tissue inhibitors of metalloproteinases; PAI-1, plasminogen activator inhibitor 1; FAK, focal adhesion kinase; MAPK, mitogen-activated protein kinase; ZO-1, zonula occludens-1; CK5, cytokeratin 5; Snail-1, Snail family of zinc-finger transcription factors 1; MT1, membrane-type 1; HIF-1α, hypoxia-inducible factor-1α; VEGF, vascular endothelial growth factor; GLUT1, glucose transporter 1; CSC, cancer stem cells; DLK1, delta like non-canonical Notch ligand 1; IL-6, interleukin 6; STAT3, Signal transducer and activator of transcription 3; α-SMA, alpha-smooth muscle actin; FAP, fibroblast activation protein alpha; TGF-β1, transforming growth factor beta 1; NOX4, NADPH oxidase 4; ROS, reactive oxygen species; ITGA5, integrin subunit alpha 5; ITGB1, integrin subunit beta 1; EMT, epithelial-mesenchymal transition; ZEB2, zinc finger E-Box binding homeobox 2; SNAI-1, Snail family transcriptional repressor 1; FOXC2, forkhead box protein C2; FN1, fibronectin 1; TGFBR1, transforming growth factor beta receptor 1; SMAD4, SMAD family member 4; CD31, cluster of differentiation 31; PPARγ, Peroxisome proliferator-activated receptor gamma; PI3K, phosphoinositide 3-kinases; AKT, protein kinase B; NF-κB, nuclear factor-κB; AMPK, AMP-activated protein kinase; HES1, hairy and enhancer of split 1; TAMs, tumor-associated macrophages; MLCK, myosin light chain kinase; ERK1, extracellular signal-regulated kinase; FZD7, Frizzled-7; NEDD9, developmentally downregulated protein 9; VIM, vimentin; KLF5, Krüppel-like factor 5; JAK, Janus kinase; CXCR4, CXC chemokine receptor-4; Slug, ces-1-related zinc finger transcription factor gene.
